# Polymolecular botanical drug of *Orthosiphon stamineus* extract (C5OSEW5050ESA) as a complementary therapy to overcome gemcitabine resistance in pancreatic cancer cells

**DOI:** 10.1016/j.jtcme.2022.10.002

**Published:** 2022-10-14

**Authors:** Ashwaq H.S. Yehya, Muhammad Asif, Amin M.S. Abdul Majid, Chern E. Oon

**Affiliations:** a Vatche and Tamar Division of Digestive Diseases, Department of Medicine, David Geffen School of Medicine at University of California Los Angeles, Los Angeles, CA, 90095, USA; b Department of Pharmacy, Faculty of Pharmacy and Alternative Medicine, The Islamia University of Bahawalpur, 63100, Pakistan; c ACRF Department of Cancer Biology and Therapeutics, The John Curtin School of Medical Research, Australian National University, 0200, Australia; d Institute for Research in Molecular Medicine (INFORMM), Unversiti Sains Malaysia, Penang, 11800, Malaysia

## Abstract

**Background and aim:**

Gemcitabine remains the cornerstone of pancreatic cancer treatment, despite exhibiting a modest effect on patient survival due to the development of drug resistance. Nuvastatic™ polymolecular botanical drug *Orthosiphon stamineus* (*O. stamineus*) is a folklore Asian herbal medicine that is used for the treatment of a variety of ailments. However, little is known about the mechanism of actions of the Nuvastatic™ polymolecular botanical drug of *O. stamineus* as a complementary therapy in resistant pancreatic cancer. It is postulated that the proprietary *O. stamineus* extract formulation (ID: C5EOSEW5050ESA) in Nuvastatic™ may sensitise resistant pancreatic cancer cells to gemcitabine. This study was conducted to assess the cytotoxic activity and synergistic effects of C5EOSEW5050ESA in gemcitabine-resistant pancreatic cancer cells.

**Experimental procedure:**

The effects of C5EOSEW5050ESA treatment on cell viability, multidrug-resistant genes, epithelial-mesenchymal transition, cellular senescence, cell death, and Notch signalling pathway were evaluated in gemcitabine-resistant Panc-1 cells.

**Results and conclusion:**

C5EOSEW5050ESA sensitised gemcitabine resistant cells towards C5EOSEW5050ESA-gemcitabine combination treatment by reducing the expression of multidrug-resistant genes and epithelial-mesenchymal transition markers in gemcitabine-resistant cells compared to the control group, possibly through the inhibition of Notch signalling. This study provides valuable insight into using C5EOSEW5050ESA as a potential complementary treatment for resistant pancreatic cancer.

## Graphical abstract

**Figure:**
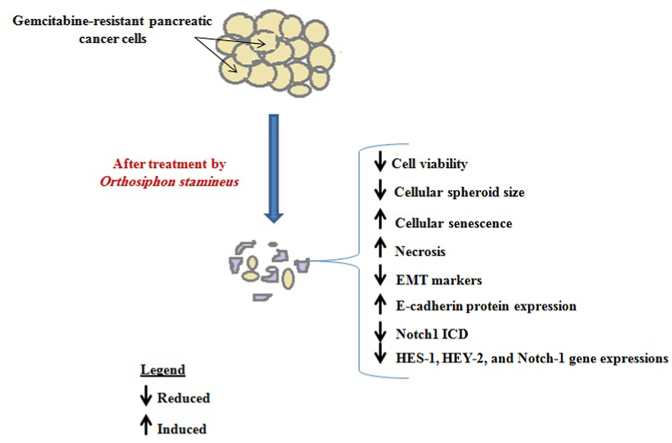

## 1 Introduction

Pancreatic cancer is one of the most fatal cancers among solid tumours because of its extensive local invasion, potency to develop drug resistance, and metastatic progression. According to GLOBOCAN 2020 estimates, pancreatic cancer was considered the 14th most widespread cancer in the world, causing 466,003 deaths (4.7% of all deaths caused by cancer) and counting 495,773 new cases in 2020.^1^ Incidentally, most patients with advanced pancreatic cancer respond poorly to chemotherapeutic agents and experience adverse drug reactions.^2^

Gemcitabine is a deoxycytidine nucleoside analogue that exerts its cytotoxic effects by inhibiting DNA synthesis and inducing cell death. However, it is only modestly effective because it often results in high systemic toxicity, including other severe side effects.^3^ Multi-drug resistance (MDR) is one of the main reasons for chemotherapy failure, leading to the recurrence of malignant tumours and, ultimately, patient relapse or death. Epithelial-mesenchymal transition (EMT) is also involved in tumour progression with metastatic expansion and the generation of tumour cells having stem cell properties that play a crucial role in resistance to cancer treatment.^4^ EMT markers are essential regulators of ABC transporters, responsible for controlling drug uptake into cancer cells to render drug insensitivity. Hence, decreasing the expression of EMT markers and their downstream components, including ABC transporters, is one of the strategies for sensitising tumour cells to therapies.^5^ Complex regulatory networks involve the Snail and ZEB (Zinc finger E-box-binding) transcriptional factors.^6^ The Notch signalling pathway plays an essential role in the development of gemcitabine resistance. The cleavage of the Notch intracellular domain (NICD) as a result of ligand binding can trigger Notch signalling. The activation of the Notch1 signalling cascade plays a pivotal role in the development of acquired resistance and gemcitabine-induced stemness in pancreatic cancer cells.^7^ The activation of Notch 1 is also known to induce EMT markers, ZEB1 and Snail-1 in pancreatic cancer cells.^8^

*Orthosiphon stamineus* (*O. stamineus*) Benth. (Lamiaceae), is commonly used asherbal tea in Europe and Southeast Asia.^9^ It is used to treat various diseases, including inflammation, bacterial infections, urinary tract infections, influenza, rheumatism, jaundice, and angiogenesis-related problems like cancer.^10^^,^^11^ Phytochemical studies have reported that the leaves of *O. stamineus* contain phenolic bioactive compounds, including rosmarinic acid, eupatorin, sinensetin, betulinic acid, pentacyclic triterpenes, ursolic acid, oleanolic acid, and β-sitosterol.^12^^,^^13^ Our previous study has demonstrated that 50% ethanol extract of *O. stamineus* inhibited angiogenesis in rat thoracic aortas and the growth of colorectal tumours in ectopic tumour nude mice model.^10^ In our recent study, *O. stamineus* significantly sensitised pancreatic (Panc-1) cancer cells towards gemcitabine byregulating MDR, EMT markers, apoptosis and the Notch signalling pathway.^8^ However, little information is available on the potential of *O. stamineus* as a complementary agent to chemotherapy in gemcitabine-resistant patients. This study aims to evaluate the cytotoxic activity and synergistic effects of a clinical trial proprietary *O. stamineus* standardised extract, C5OSEW5050ESA *(*Nuvastatic™) on its ability to reverse MDR in gemcitabine resistant pancreatic cancer cells. The drug substance in Nuvastatic™ is Lanctos75™ (C5EOS5050ESA), an ethanolic extract of a unique genotype of *Orthosiphon stamineus*/aristatus leaves, a mixture of polyphenols and other components of this plant species. Nuvastatic™ has recently completed phase 2/3 clinical studies (clinicaltrial.gov) for cancer fatigue in cancer patients with solid tumours stage I–IV receiving chemotherapy and/or radiotherapy ^82^. The drug was shown to significantly reduce fatigue and improve the quality of life of test subjects.

## 2 Materials and methods

### 2.1 Cell lines

Panc-1 pancreatic cancer cell line was purchased from ATCC (CRL-1469™). Panc-1 Gem cells were derived from Panc-1 cells through injections into SCID mice for three months and subsequent treatment with 50 mg/kg of gemcitabine as reported by Oon et al., 2015.^14^ Similarly, Panc-1 PBS cells were derived from Panc-1 cells through injections into SCID mice for three months, but with subsequent administration of 1 ml/kg of phosphate buffer saline (PBS).^14^ The passage number of cells was from 2 to 6. The Stockholm North Ethical Committee on Animal Experiments approved the animal experiments and the Karolinska animal ethics review boards (NR166/12). The normal colon epithelial cell line (CCD841) was purchased from ATCC (CRL-1790). All cells were maintained in Dulbecco's Modified Eagle Medium (DMEM) (Thermo Scientific, USA), supplemented with 10% fetal bovine serum (Biowest, USA) and 100 units/ml penicillin-streptomycin (Nacalai Tesque, USA). The cells were kept in a humidified 5% carbon dioxide (CO_2_) environment in cell culture incubators set at 37 °C.

### 2.2 Plant materials and compounds

C5EOSEW5050ESA was purchased from NatureCeutical Sdn Bhd, Malaysia. Quantitative determination of flavonoids (rosmarinic acid, eupatorin, and sinesitin) was previously performed using the HPLC method as reported in Yehya et al., 2020.^8^ Gemcitabine (Catalogue No. S1149) was obtained from Selleckchem, USA. Rosmarinic acid (Catalogue No: 536954) and eupatorin (Catalogue No: E4660) was purchased from Sigma-Aldrich, USA. Sinesitin (Catalogue No: P201) was purchased from IndofineUSA. All the compounds and C5EOSEW5050ESA were dissolved in sterile deionized water.

### 2.3 Cell viability assay

The MTT assay was performed as described by Yehya et al., 2020^8^ to measure the IC_50_ (50% inhibitory concentration) of *O. stamineus* (C5EOSEW5050ESA), gemcitabine, rosmarinic acid, eupatorin, and sinesitin respectively. Briefly, the cells were seeded in a 96-wells plate and incubated at 37 °C in a humidified atmosphere of 5% carbon dioxide (CO_2_) for 24 h. Different doses of gemcitabine (0, 50, 100, 150, 200, 250, 300, 350, and 400 μM), *O. stamineus* (0, 15.6, 31.25, 62.5, 125, 250, 500, 1000, 2000 μg/ml), eupatorin (0, 25, 50, 75, 100, 125, 150, 175, 200 μM), sinesitin (0, 25, 50, 75, 100, 125, 150, 175, 200 μM), and rosmarinic acid (0, 2.5, 5, 10, 20, 40, 80, 160, 320 μM) were tested on different cell lines. The plate was incubated at 37 °C in a humidified atmosphere of 5% CO_2_ for 72 hat 37 °C. MTT reagent was added and then incubated for 4–8 h. The optical density was measured using a microplate reader (Tecan, Switzerland) at 570 nm and with the reference wavelength at 620 nm.

### 2.4 Hanging drop spheroid assay

Drops (20 μl) of medium containing 50,000 cells were seeded onto 100 mm Petri dish lids and inverted. The hanging drops were incubated for 72 h to promote sedimentation, and the resulting aggregate cells (spheroids) were harvested cautiously using pipette tips and then introduced into a 48-well plate coated with 1% agar (20 μl/drop) and 180 μl of the medium was added per well. The spheroids were treated with different concentrations of C5EOSEW5050ESA and eupatorin. Images of the spheroids were taken on days 0, 1, 3, 5, and 7. The diameter of the spheroids was calculated using ImageJ.^15^

### 2.5 Cellular senescence assay

The senescence b-Galactosidase staining kit (Cell Signaling Technology, USA) was used to detect b-galactosidase activity, according to the manufacturer's instructions. Cells were seeded into 6-well plates and incubated at 37 °C in a humidified atmosphere of 5% CO_2_ for 24 h to enhance cell attachment. The media were gently removed and replaced with fresh media containing different treatment concentrations and incubated for 72 h. At least 300 cells per treatment condition, cells were analysed for cellular senescence (ratio of blue cells over the total number of cells) under a microscope, as previously described 14.

### 2.6 Detection of cell cycle arrest

The cancer cells were prepared according to the manufacturer's instructions (Life Technologies, USA). The cells were treated and incubated for 72 h in a 6-well plate. The cells were then trypsinised and washed in cold 1x PBS, followed by fixation in 1 ml of 70% ethanol for 30 min. Cells werecentrifuged, washed with cold PBS, and centrifuged at 45×*g* for 10 min. Finally, the cell pellet was stained with propidium iodide (PI) solution (Life Technologies, USA). Stained cells were analysed using a BD FACSCalibur (BD Biosciences, USA).

### 2.7 Quantitative real-time PCR

RNA was harvested from 5 × 10^4^ cells/ml after 72 h of treatment using T GENEzol™ Reagent (Geneaid, Taiwan) and reversed to cDNA using a high-capacity cDNA reverse transcription kit (Applied Biosystems, Warrington). The method of Real-time PCR quantitative (qPCR) was described by Yehya et al., 2020.^8^ The cDNA samples were programmed through 40 cycles of amplification at 95 °C for 15 s and 60 °C for 1 min. Genes werenormalised to the Glyceraldehyde 3-phosphate dehydrogenase (GAPDH) housekeeping gene. The following primer sets were used for qPCR, GAPDH: F′ 5-ACCCACTCCTCCACCTTTGA-3 and R′ 5- CTGTTGCTGTAGCCAAATTCGT -3. MDR-1-F′ 5-CCCATCATTGCAATAGCAGG-3 and MDR-1-R′ 5-GTTCAAACTTCTGCTCCTGA-3. MRP-4-F′ 5- GGATCCAAGAACTGATGAGTTAAT -3 and MRP-4-R′ 5-TCACAGTGTGTCTCGAAAATAG-3. Snail-1-F′ 5-TCGGAAGCCTAACTACAGCGA-3 and Snail-1-R′ 5-AGATGAGCATTGGCAGCGAG-3. ZEB-1-F′ 5-TTACACCTTTGCATACAGAACCC-3 and ZEB-1-R′ 5-TTTACGATTACACCCAGACTGC-3. HES-1-F′ 5-AAGAAAGATAGCTCGCGGCA-3 and HES-1-R′ 5-AAACACCTTAGCCGCCTCTC-3. HEY-2-F′ 5-GTTGCGGCGTGGGAAAGAG-3 and HEY-2-R′ 5-GTGTGGGTCAAAGTAGCCTTTA-3. Notch-1-F′ 5-GACAACGCCTACCTCTGCTT-3 and Notch-1-R’ 5-ACTTGTACCCGTTGAGGCTG-3.

### 2.8 Western blot

Protein lysates were extracted from cultured cells 72 h post-treatment using RIPA lysis buffer (Nacalai Tesque, USA) according to the manufacturer's instructions. The protein samples were quantified using the NanoDrop ND-1000 spectrophotometer (ThermoScientific, USA) at 280 nm wavelengthand loaded at 40 mg/well into10-15%bisacrylamide gel (Nascalai Tesque, Japan). Protein samples were then transferred from the gel onto the Immobilon-polyvinylidene fluoride transfer membrane (Millipore, Watford). Blocking solution (5% milk powder and 0.1% Tween-20 in PBS) was used to immerse the membrane for 1 h at room temperature. Then, the membrane was probed with primary antibodies (Notch 1 ICD, Vimentin, E-Cadherin, and Activated caspase-3 (Santa Cruz, USA); Cleaved PARP (Cell Signalling Technology, USA); b-Actin (Sigma Aldrich, USA)) overnight at 4 °C. The membrane was washed in PBST three times (10 min each) and probed with appropriate HRP-conjugated secondary antibodies. The protein bands were detected using Chemi-Lumi one super detection reagents (Nacalai Tesque, USA) and visualised using the C-Digit blot scanner (Lincoln, Nebraska, USA). Image J was used to measure the relative density of each peak with the size and intensity of each band on the blot and normalised to the loading control (b-Actin). Analyses were performed on data from three independent experiments.

### 2.9 Cell death detection

Cell death was detected by flow cytometry using the Annexin V-FITC Apoptosis Detection Kit (eBioscience, Austria). Cells were seeded at a density of 50,000 cells in 10% growth medium and incubated at 37 °C in a humidified atmosphere of 5% (v/v) CO2 to allow cell attachment. Cells were then treated with C5EOSEW5050ESA for 72 h. Cells were trypsinised, centrifuged at 10×*g* for 5 min, and further rinsed in 1x PBS with gentle shaking prior to staining with propidium iodide (PI) and annexin V for 10–15 min according to the manufacturer's instructions. Etoposide was used as the positive control for apoptosis and necrosis. After incubation (10–15 min), cells were immediately subjected to flow cytometry analysis using BD FACSCalibur (BD Biosciences, USA). The experiment was carried outas independent triplicates.

### 2.10 Statistical methods

The Prism (GraphPad, USA) and graphing software Excel (Microsoft, USA) were used for statistical analysis. Data was presented as mean ± S.D., and statistics were performed using one-way analysis of variance (ANOVA) with the Tukey's Honest Significant Difference (HSD) posthoc test to compare the mean values among three or more data sets. A value *p* < 0.05 was considered significant as compared to the respective control group of all groups.

## 3 Results and discussion

### 3.1 Panc-1 gem cells demonstrated resistance to gemcitabine and C5EOSEW5050ESA partially sensitised resistant cells to gemcitabine treatment with increasing doses

**Fig. 1:**
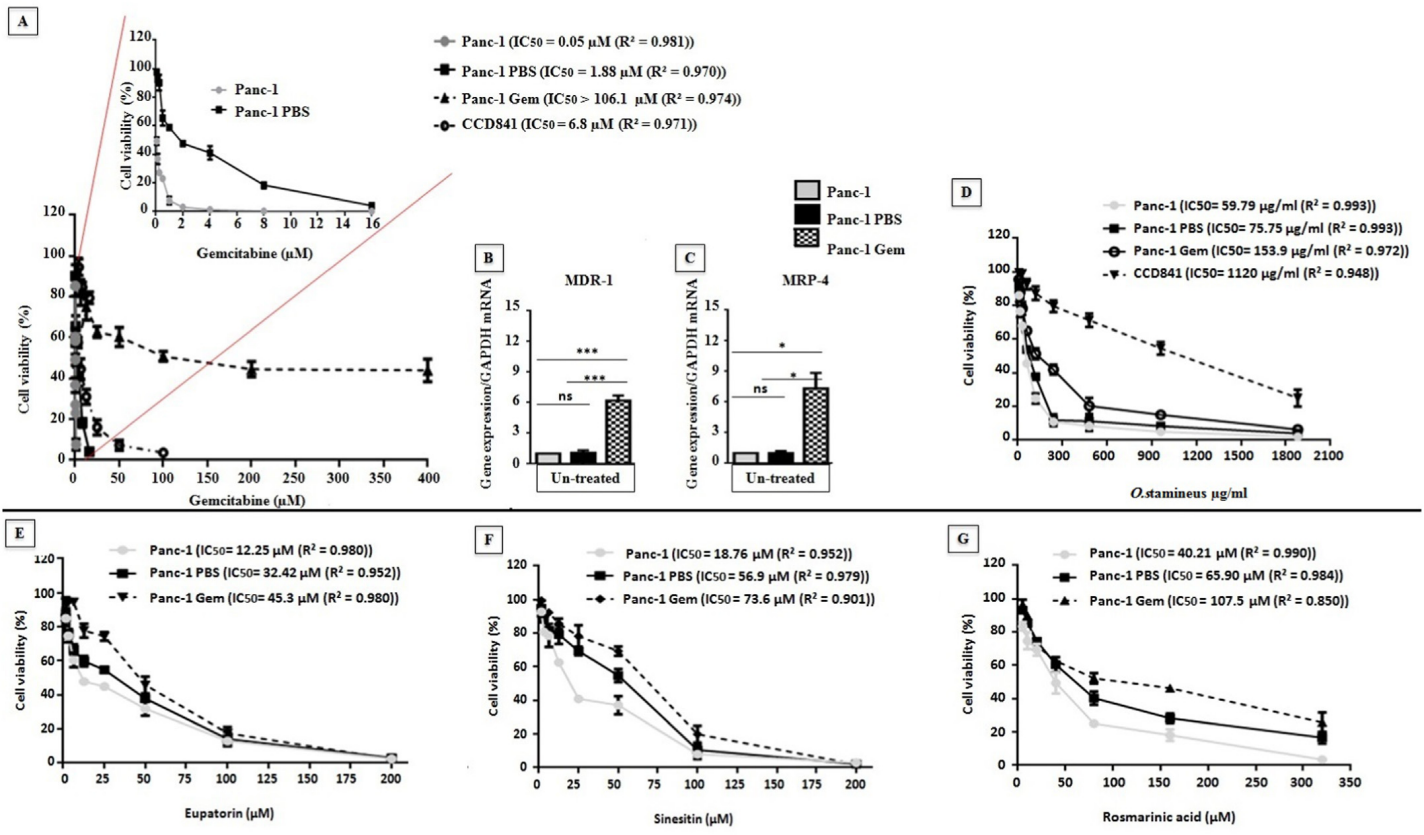
Effect of gemcitabine, *O. stamineus* (C5EOSEW5050ESA), eupatorin, sinesitin, and rosmarinic acid on the cell viability of Panc-1, Panc-1 PBS, Panc-1 Gem, and CCD841 normal colon epithelial cells and multi-drug-resistant genes. A) Panc-1 PBS showed less sensitivity towards gemcitabine compared to Panc-1 cells. B) Panc-1- Gem demonstrated established resistance towards gemcitabine, and C) Panc-1 Gem cells showed increased MDR-1 and MRP-4 gene expressions compared to untreated Panc-1 PBS and Panc-1 cells. D) *O. stamineus* reduced the cell viability of all three cell lines with increasing concentration. At concentrations of 480 μg/ml, *O. stamineus* did not show a further reduction of Panc-1 Gem cell viability compared to that of the same level as Panc-1 PBS and Panc-1. E) Eupatorin reduced cell viability of all three cell lines with increasing concentration. Eupatorin at 50 μM and 100 μM reduced cell viability of Panc-1 Gem to the same level as Panc-1PBS. F) Sinesitin reduced cell viability of all three cell lines with increasing concentration. Sinesitin at 25 μM, 50 μM, and 100 μM reduced cell viability of Panc-1 Gem compared to Panc-1 PBS. G) Rosmarinic acid reduced the cell viability of all three cell lines with increasing concentration. At lower concentrations of 25 μM, rosmarinic acid showed reduced cell viability of Panc-1 Gem to the same level to Panc-1 PBS. However, rosmarinic acid at 50 μM demonstrated fewer efficacies in reducing Panc-1 gem cell viability compared to Panc-1 PBS. Error bars represent SD. Statistics analysis (ns = not significant; ∗*P* < 0.05; ∗∗*P* < 0.01; ∗∗∗*P* < 0.001, One way ANOVA with Tukey's HSD post-hoc test, n = 3 independent experiments) using GraphPad Prism 6.0 software.

We confirmed Panc-1 Gem to display established resistance towards gemcitabine compared to Panc-1 PBS (vehicle control) and Panc-1 cells through MTT assay and P-glycoprotein gene expression analyses (Fig. 1 A).Panc-1 and Panc-1 PBS demonstrated different IC_50_ in response to gemcitabine treatment at 0.05 μM and 1.88 μM respectively (Fig. 1A). Panc-1 Gem cells showed an increase in MDR-1 and MRP-4 genes expression compared to Panc-1 PBS and Panc-1 cells (Fig. 1B and C). The IC_50_ value of gemcitabine for CCD841 normal colon epithelial cells was 6.8 μM. All Panc-1, Panc-1 PBS, and Panc-1 Gem cells showed a reduction in cell viability in response to C5EOSEW5050ESA in increasing concentrations (Fig. 1D). The IC_50_ values of C5EOSEW5050ESA for Panc-1, Panc-1 PBS, and Panc-1 Gem cells were 59.79 μg/ml, 75.75 μg/ml, and 153.9 μg/ml respectively. The IC_50_ value of C5EOSEW5050ESA for CCD841 normal colon epithelial cells was 1120 μg/ml. C5EOSEW5050ESA reduced Panc-1 Gem resistant cell viability more than CCD841 cells (Fig. 1A). At 480 μg/ml, C5EOSEW5050ESA showed no further reduction of Panc-1 Gem cell viability compared to Panc-1 PBS or Panc-1 cells (Fig. 1A).

### 3.2 Eupatorin reduced the cell viability of resistant cells compared to sinesitin and rosmarinic acid

Eupatorin, sinesitin, and rosmarinic acid are the main bioactive compounds of C5EOSEW5050ESA, hence these components were tested to assess their efficacy as a single compound alongside C5EOSEW5050ESA polymolecular drug. Eupatorin, sinesitin, and rosmarinic acid reduced the cell viability of Panc-1 Gem to the same level as Panc-1 PBS cells at 50, 75, and 100 μM respectively (Fig. 1E, F, and 1G). The IC_50_ values of eupatorin in Panc-1, Panc-1 PBS and Panc-1 Gem were12.25 μM, 32.42 μM, and 45.30 μM, respectively. However, the IC_50_ values of sinesitin inPanc-1, Panc-1 PBS and Panc-1 Gem were 18.76 μM, 56.9 μM, and 73.6 μM, respectively, whereas the IC_50_ of rosmarinic acid for Panc-1 was 40.21 μM, Panc-1 PBS; 65.90 μM, and Panc-1 Gem; 107.5 μM. Eupatorin was superior at reducing Panc-1 Gem cell viability at a lower IC_50_compared to sinesitin or rosmarinic acid (Fig. 1E). Thus, eupatorin was selected to compare the efficacy between C5EOSEW5050ESA and a single compound enriched in the extract. Eupatorin demonstrated a better effect at reducing the viability of Panc-1 Gem cells at a lower IC_50_ compared to sinesitin and rosmarinic acid (Fig. 1E, F, and G).

### 3.3 C5EOSEW5050ESA and eupatorin reduced the viability of gemcitabine-resistant Panc-1 spheroids

**Fig. 2:**
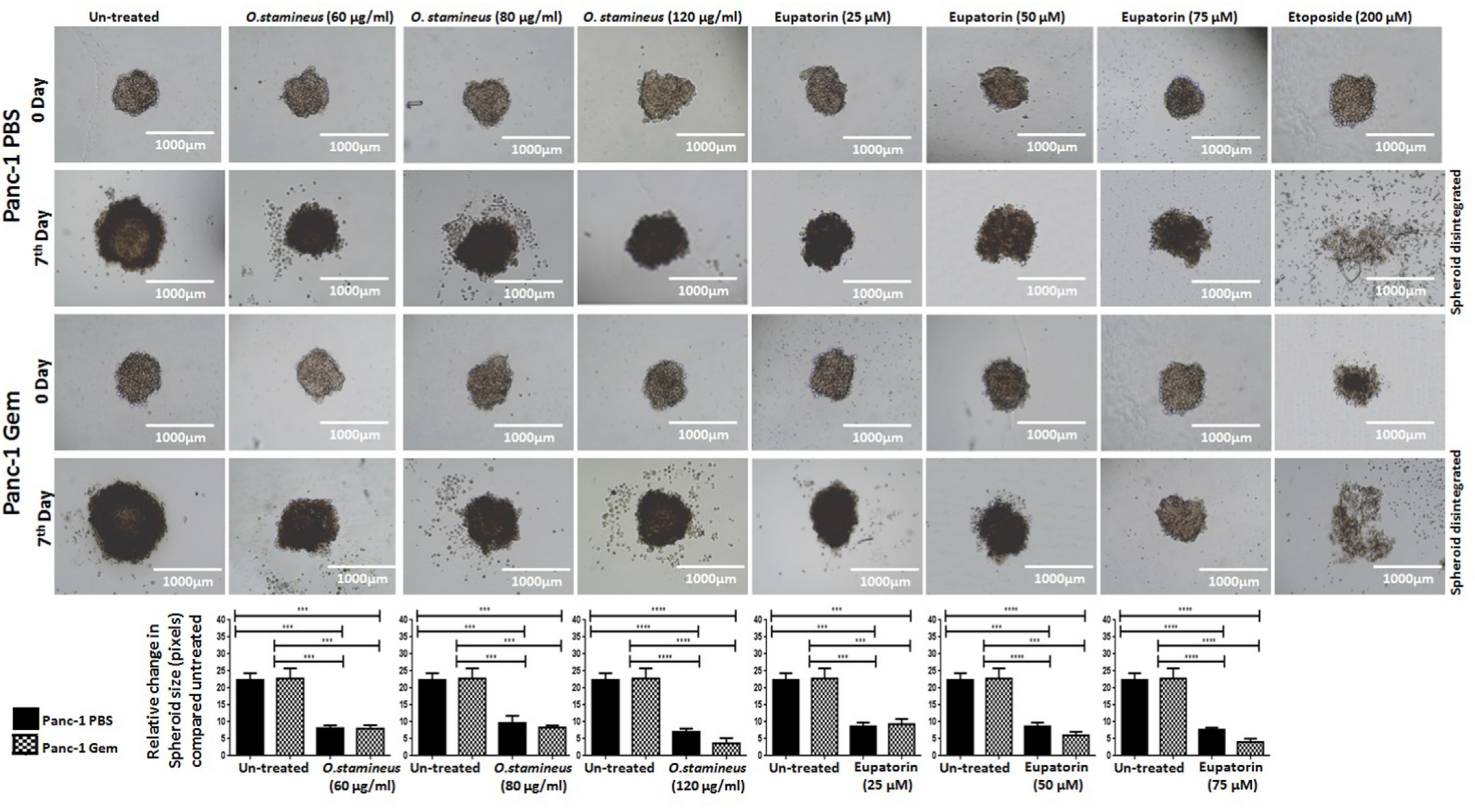
Anti-tumour aggregation of *O. stamineus* (C5EOSEW5050ESA) and eupatorin on Panc-1 PBS and Panc-1 Gem cellular spheroids in the hanging drop assay after seven days of treatment. The cells were treated with different concentration of *O. stamineus*, 60, 80, and 120 μg/ml, eupatorin, 25, 50, and 75 μM, G) and etoposide (Positive control), 200 μM treatments. Analysis of spheroid diameter using ImageJ indicated that *O. stamineus* at 120 μg/ml induced the greatest disruption of the spheroids in Panc-1 Gem cells compared to Panc-1 PBS. Statistics analysis (∗*P* < 0.05; ∗∗*P* < 0.01; ∗∗∗*P* < 0.001, One way ANOVA with Tukey's HSD post-hoc test, n = 3 independent experiments) using GraphPad Prism 6.0 software.

To investigate the effect of a single compound or C5EOSEW5050ESA on tumour viability, we employed the spheroid tumour model, which often consists of cells in different proliferative and metabolic states, thus mimicking the native tissue more accurately. C5EOSEW5050ESA and eupatorin significantly reduced spheroid size compared to untreated Panc-1 PBS and Panc-1 Gem spheroids (Fig. 2). C5EOSEW5050ESA at 120 μg/ml reduced the size of spheroids accompanied by cell debris compared to untreated Panc-1 PBS spheroids (Fig. 2). Similarly, eupatorin at 75 μM significantly shrank Panc-1 Gem spheroids compared to Panc-1 PBS spheroids.

### 3.4 C5EOSEW5050ESA promoted cellular senescence in gemcitabine-resistant cells

**Fig. 3:**
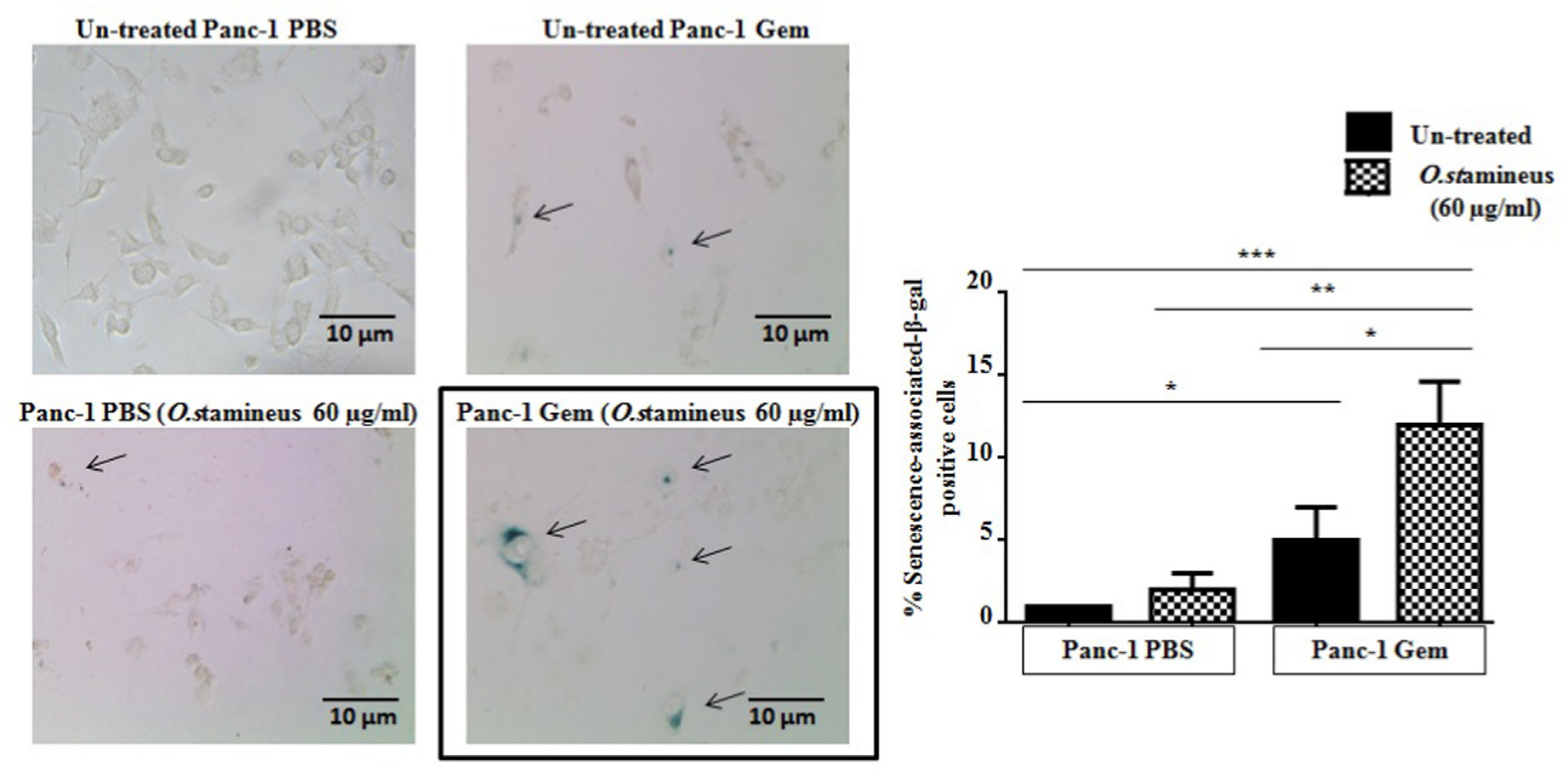
*O. stamineus* (C5EOSEW5050ESA) induced cellular senescence in resistant cells. Senescence-associated-β-gal activity was measured on Day 6 and scored as the percentage ofsenescence-associated-β-gal positive cells (blue) over total 500 cells (100 x magnifications). *O. stamineus* at 60 μg/ml induced cellular senescence in Panc-1Gem compared to Panc-1PBS. Error bars represent SD. Statistics analysis (∗*P* < 0.05; ∗∗*P* < 0.01; ∗∗∗*P* < 0.001, One way ANOVA with Tukey's HSD post-hoc test, n = 3 independent experiments) using GraphPad Prism 6.0 software.

To determine the mechanism of action involved in C5EOSEW5050ESA-induced sensitisation of gemcitabine-resistant cells, we performed assays to determine their effects on senescence, cell death, as well as epithelial-mesenchymal transition (EMT) and multi-drug resistance gene regulation. C5EOSEW5050ESA alone did not affect senescence compared to untreated Panc-1 PBS cells (Fig. 3). However, C5EOSEW5050ESA significantly induced cellular senescence in Panc-1 Gem cells compared to untreated Panc-1 Gem cells. Panc-1 Gem showed the induction of cellular senescence compared to untreated Panc-1 PBS (Fig. 3), however, C5EOSEW5050ESA significantly induced cellular senescence in Panc-1 Gem cells compared to untreated Panc-1 Gem cells.

### 3.5 C5EOSEW5050ESA had no effect on cell cycle distribution in gemcitabine-resistant cells

**Fig. 4:**
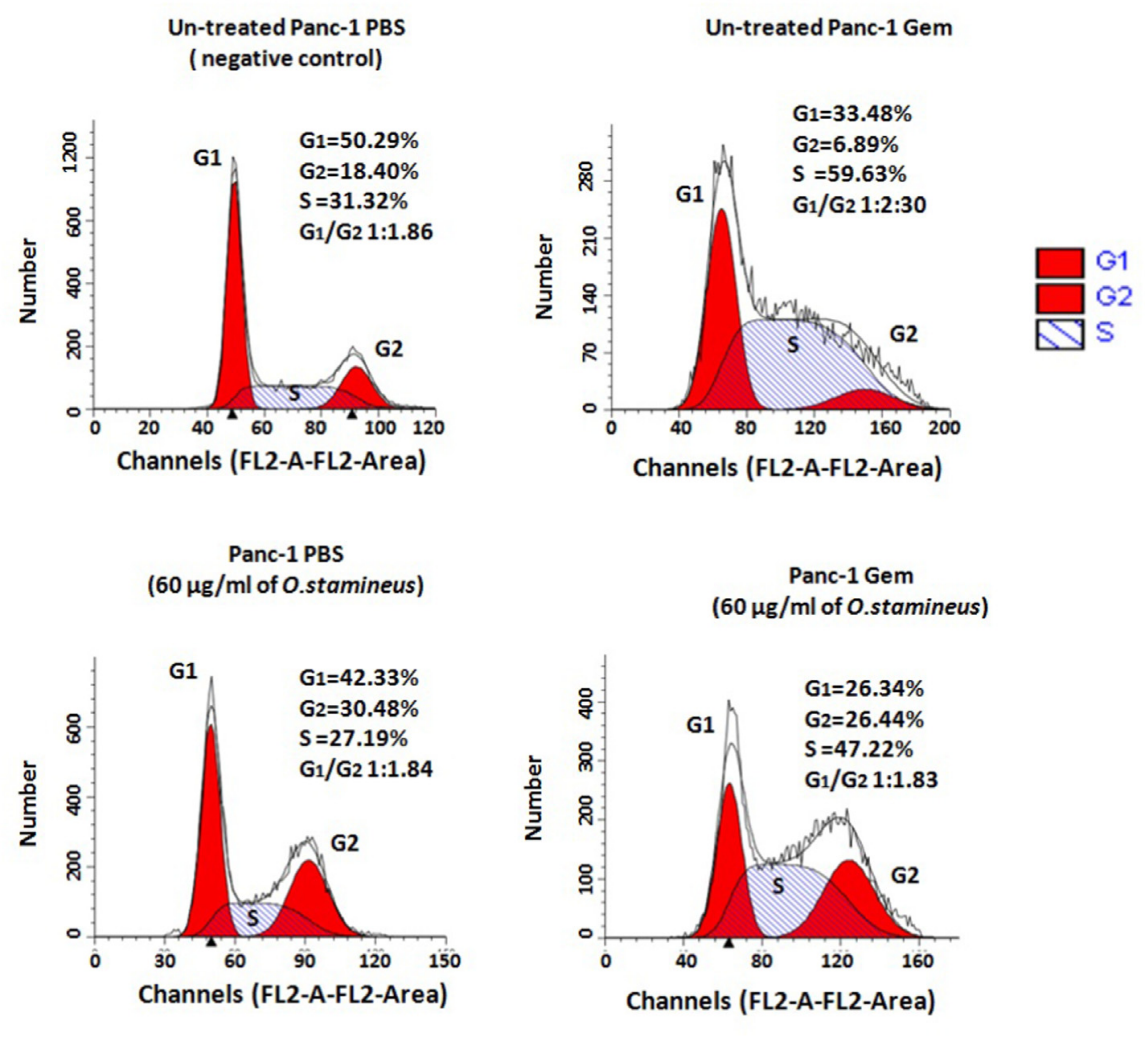
Effect of *O. stamineus* (C5EOSEW5050ESA) on cell cycle distribution of Panc-1 cells at 72 h post-treatment. *O. stamineus* showed no additional effect on cell cycle arrest in Panc-1 gemcitabine-resistant cells. The figure is representative of 3 independent experiments. G1: Gap 1, G2: Gap2, S: Synthesis.

To determine the cytostatic potential of C5EOSEW5050ESA, flow cytometry was performed to evaluate its effect on cell cycle distribution. In comparison with untreated Panc-1 PBS, Panc-1 Gem was arrested at the S phase (Fig. 4). C5EOSEW5050ESA induced G2 arrest in Panc-1 PBS. However, C5EOSEW5050ESA did not show any additional effect on cell cycle arrest in Panc-1 Gem cells (Fig. 4).

### 3.6 C5EOSEW5050ESA decreased the expression of multi-drug resistance genes in gemcitabine-resistant cells compared to eupatorin

**Fig. 5:**
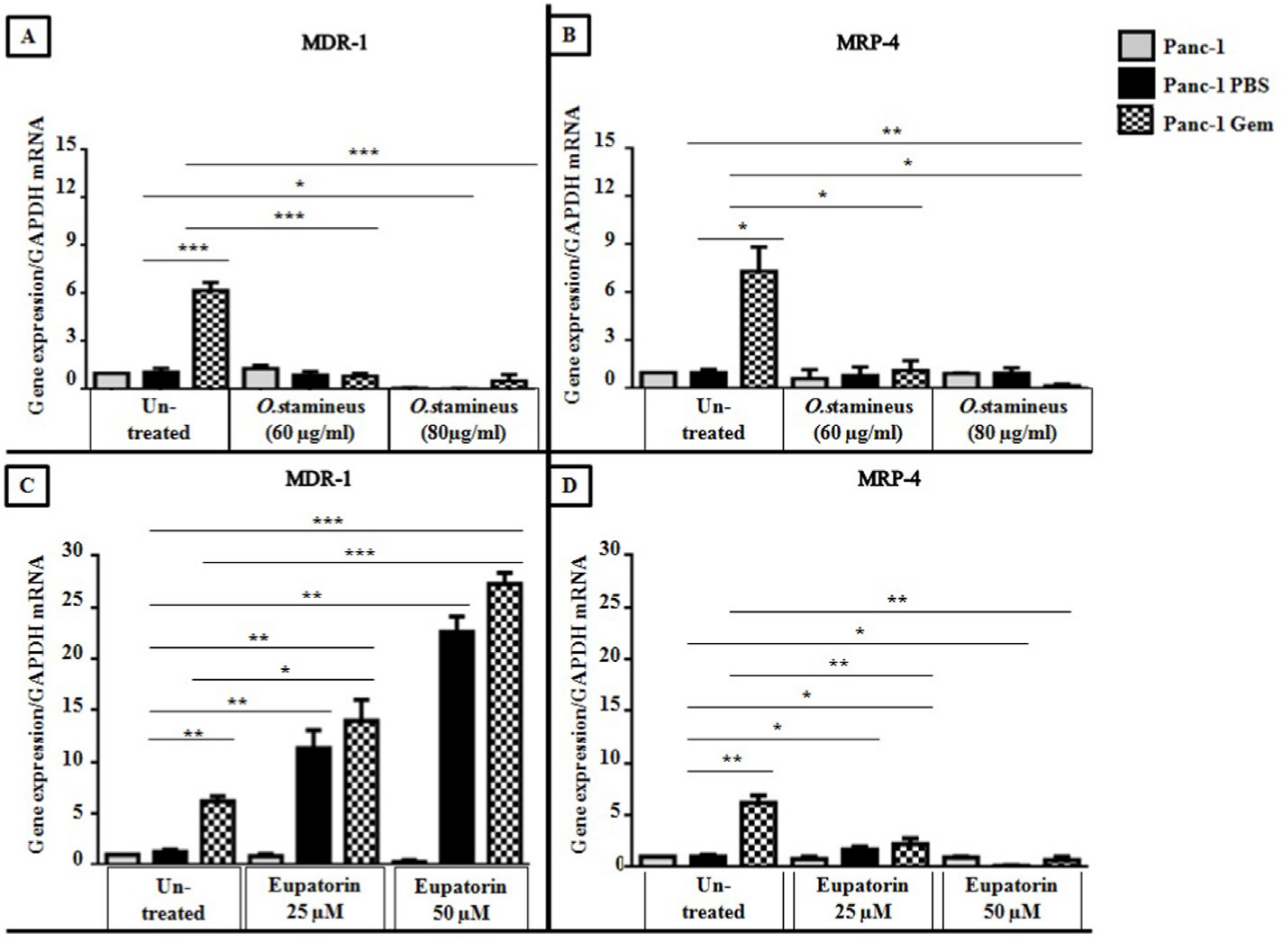
Effects of *O. stamineus* (C5EOSEW5050ESA) and eupatorin treatments on the expression of multi-drug resistance 1 (MDR-1) and multi-drug resistance protein 4 (MRP-4) genes in Panc-1, Panc-1 PBS, and Panc-1 Gem cells 72 h post-treatment. A and B (*O. stamineus* treatment); C and D (eupatorin treatment). Error bars represent SD. Statistics analysis (∗*P* < 0.05; ∗∗*P* < 0.01; ∗∗∗*P* < 0.001, One way ANOVA with Tukey's HSD post-hoc test, n = 3 independent experiments) using GraphPad Prism 6.0 software.

To confirm gemcitabine resistance, the expression of MDR-1 and MRP-4 multi-drug resistance genes were evaluated in Panc-1 Gem, Panc-1 PBS, and Panc-1 cells. Panc-1 Gem cells showed an increase in MDR-1 and MRP-4 gene expression compared to Panc-1 PBS cells (Fig. 5). C5EOSEW5050ESA treatments at 60 and 80 μg/ml showed no increase in MDR-1 gene expression compared to untreated cells in Panc-1 PBS and Panc-1 (Fig. 5A). At both concentrations, C5EOSEW5050ESA significantly reduced the expression of MDR-1 and MRP-4 genes in Panc-1 Gem by 5 ± 0.57 and 8 ± 0.98 folds compared to untreated Panc-1 Gem, however, no significant difference in gene expression of MDR-1 and MRP-4 was observed between Panc-1 Gem and Panc-1 PBS cells when both groups were treated with C5EOSEW5050ESA (Fig. 5A and B). Furthermore, no substantial decrease in the expression of MDR-1 and MRP-4 genes in Panc-1 Gem was observed when cells were treated with 80 μg/ml C5EOSEW5050ESA. C5EOSEW5050ESA at lower concentrations (60 μg/ml) had the same effect as with higher concentrations that reduced gemcitabine efflux from Panc-1 Gem by down-regulating the MDR-1 and MRP-4 gene expressions.

Eupatorin at 25 and 50 μM further increased MDR-1 gene expression (5 ± 0.57 and 16 ± 0.32 folds respectively) compared to the untreated group in Panc-1 PBS. As a result, Panc-1 Gem exhibited increased MDR-1 gene expression compared to Panc-1 PBS (Fig. 5C). Similarly, MDR-1 gene expression was also increased in Panc-1 gem (8 ± 0.99 and 21 ± 0.81 folds respectively), compared to the untreated group when treated with eupatorin at 25 and 50 μM, respectively. However, eupatorin at 25 μM and 50 μM significantly reduced MRP-4 gene expression (6 ± 0.03 and 7 ± 0.05 folds respectively) in Panc-1 Gem cells compared to untreated cells (Fig. 5D).

### 3.7 C5EOSEW5050ESA reversed epithelial-mesenchymal transition (EMT) in gemcitabine-resistant cells

**Fig. 6:**
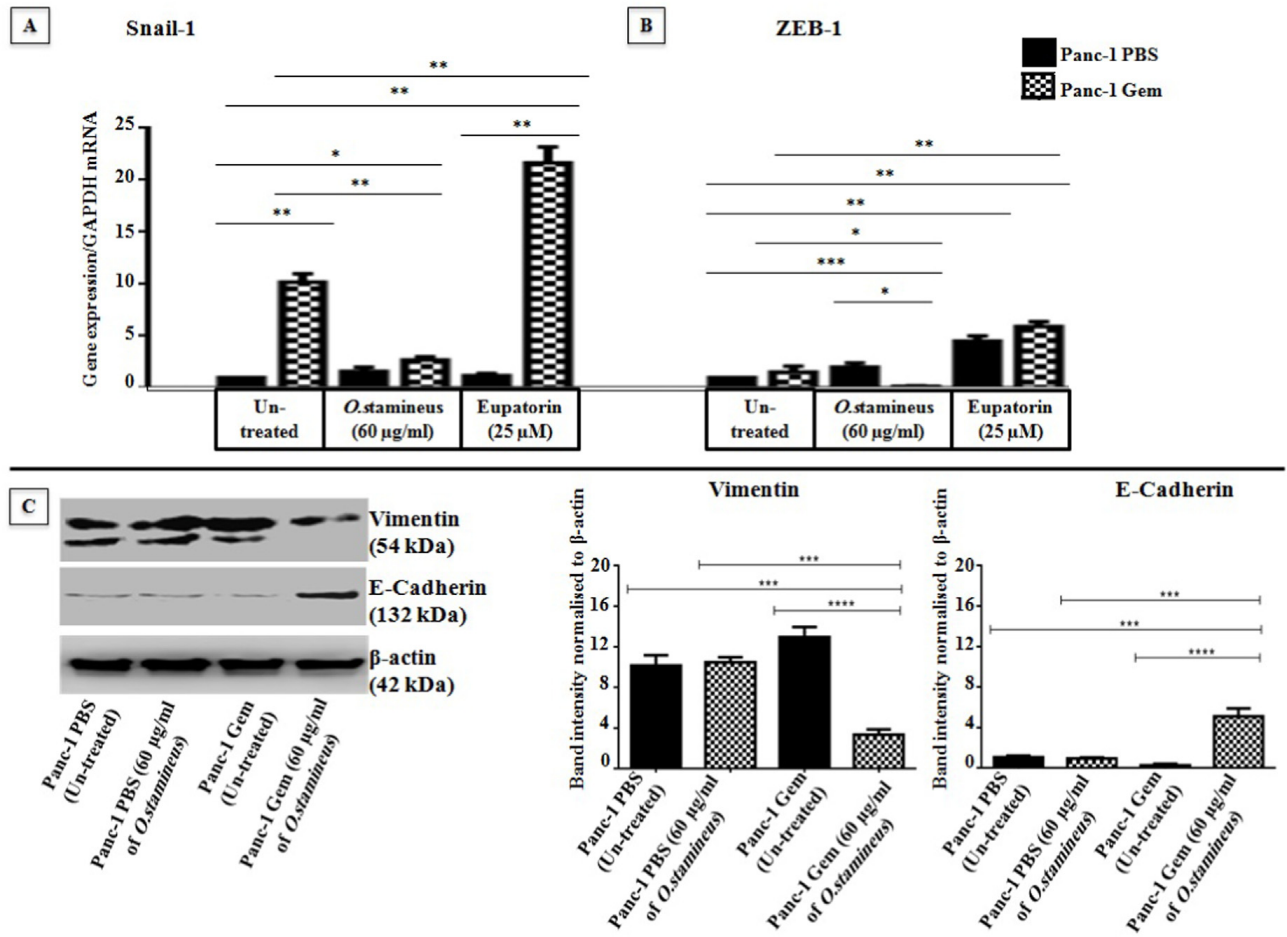
Effects of *O. stamineus* (C5EOSEW5050ESA) on the expression of epithelial-mesenchymal transition (EMT) markers on Panc-1 PBS and Panc-1 Gem cell lines for 72 h. A) *O. stamineus* treatment at 60 μg/ml and eupatorin at 25 μM on the expression of Snail-1 gene. B) Effects of *O. stamineus* treatment at 60 μg/ml and eupatorin at 25 μM on the expression of ZEB-1 gene. C) The effect of *O. stamineus* on the expression of vimentin and E-Cadherin proteins in Panc-1 PBS and Panc-1 Gem. Error bars represent SD. Statistics analysis (∗*P* < 0.05; ∗∗*P* < 0.01; ∗∗∗*P* < 0.001; ∗∗∗∗*P* < 0.0001 One way ANOVA with Tukey's HSD post-hoc test, n = 3 independent experiments) using GraphPad Prism 6.0 software.

EMT is a phenomenon known in mediating resistance to chemotherapies. Snail-1 and ZEB-1 are two transriptions factors indicated in EMT. C5EOSEW5050ESA significantly reduced the expressions of Snail-1 in Panc-1 Gem cells by 7 ± 0.05 compared to Panc-1 Gem (Fig. 6A and B). ZEB-1 gene expression remained unchanged in untreated Panc-1 Gem cells compared to untreated Panc-1 PBS. However, C5EOSEW5050ESA decreased ZEB-1 expression (1 ± 0.03 and 2 ± 0.02) in Panc-1 Gem compared to untreated Panc-1 PBS and Panc-1 Gem respectively. C5EOSEW5050ESA did not significantly reduce Snail-1 expression (1 ± 0.24) in Panc-1 PBS compared to its untreated group (Fig. 6A). Besides, C5EOSEW5050ESA reduced vimentin protein expression in Panc-1 Gem (5 ± 0.82 and 9 ± 0.37) compared to untreated Panc-1 PBS and treated Panc-1 Gem cells respectively and induced E-cadherin protein expression in Panc-1 Gem by 4 ± 0.63 and 4.5 ± 0.82 compared to untreated Panc-1 PBS and Panc-1 Gem respectively (Fig. 6C and D). The expression of vimentin mesenchymal protein was not reduced in Panc-1 PBS cells when treated with C5EOSEW5050ESA (Fig. 6C). In contrast, eupatorin up-regulated ZEB-1 gene expression by 2 ± 0.43 Panc-1 PBS when compared with untreated. Eupatorin also up-regulated Snail-1 and ZEB-1 expression (20 ± 0.81 and 12 ± 0.93 respectively) in Panc-1 Gem compared to untreated Panc-1 PBS and Panc-1 Gem (Fig. 6A and B).

### 3.8 C5EOSEW5050ESA inhibited the Notch signalling pathway in gemcitabine-resistant cells

**Fig. 7:**
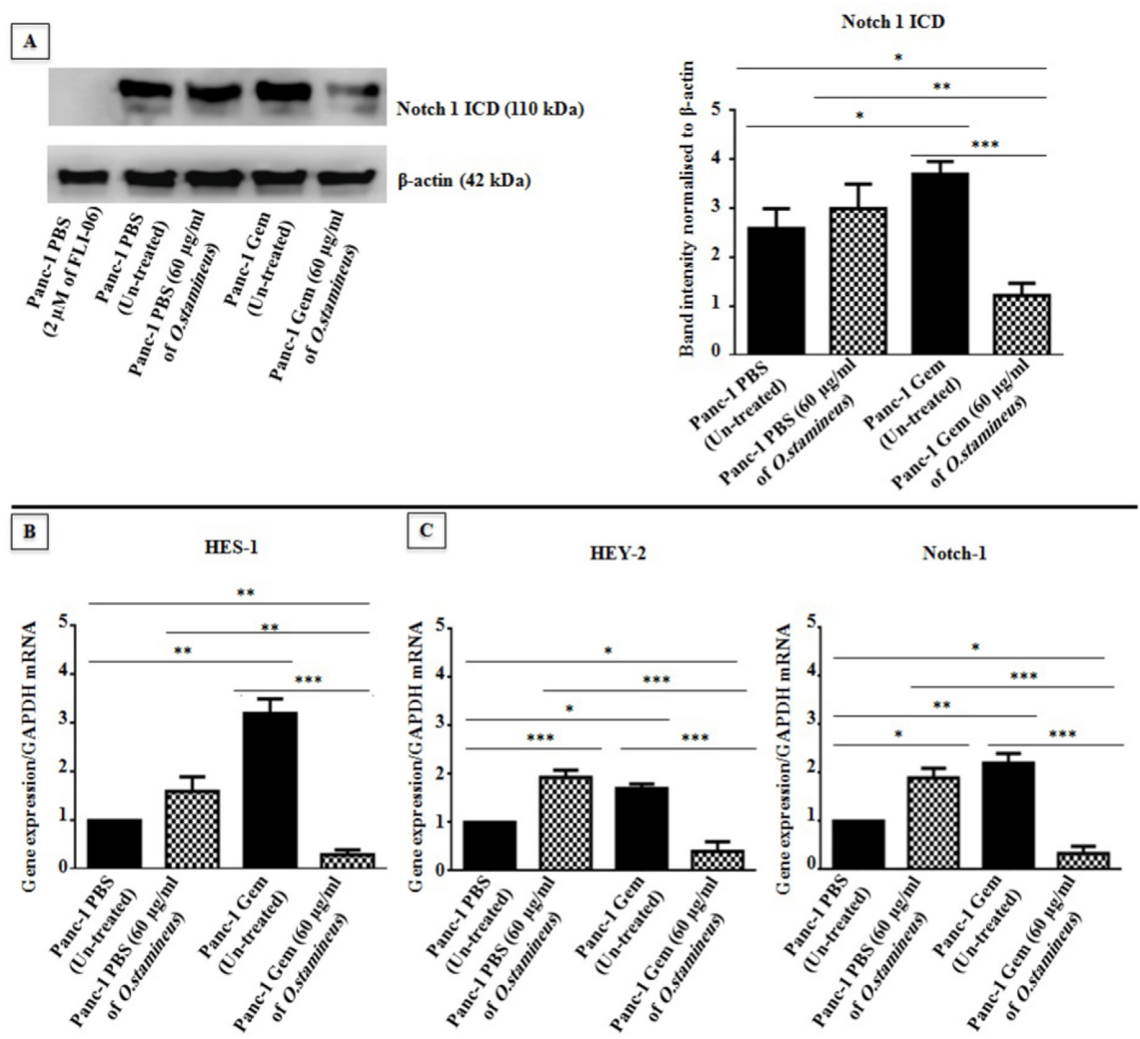
Protein expression profile of Notch 1 ICD and gene expression profile of HES-1, HEY2, and Notch-1 in resistant cells post-treatment with or without *O. stamineus* (C5EOSEW5050ESA) for 72 h. A) Notch-1 ICD protein expression was investigated in Panc-1 cells through Western blot. B) HES-1 gene expression in gemcitabine-resistant cells. C) HEY-1 gene expression in gemcitabine-resistant cells. D) Notch-1 gene expression in gemcitabine-resistant cells. FLI-06 (Notch inhibitor) was used as a negative control for blockage of Notch signalling. Error bars represent SD. Statistics analysis (∗*P* < 0.05; ∗∗*P* < 0.01; ∗∗∗*P* < 0.001, One way ANOVA with Tukey's HSD post-hoc test, n = 3 independent experiments) using GraphPad Prism 6.0 software.

Notch signaling is implicated in cancer therapeutic resistance. In this study, Panc-1 Gem increased the Notch 1 intracellular domain (Notch1ICD) expression (I.2 ± 0.65) compared to Panc-1 PBS, indicating the activation of Notch signalling through the Notch 1 receptor (Fig. 7). C5EOSEW5050ESA did not affect Notch1ICD protein expression in Panc-1 PBS when compared to untreated Panc-1 PBS. Nevertheless, C5EOSEW5050ESA significantly reduced Notch 1ICD protein expression (1.6 ± 0.42 and 2.6 ± 0.36) in Panc-1 Gem cells compared to untreated Panc-1 PBS and Panc-1 Gem respectively (Fig. 7A). Notch inhibitor (FLI-06) was used as a positive control. Also, Panc-1 Gem significantly induced HES-1 (2 ± 0.89), HEY-2 (0.8 ± 0.68), and Notch 1 (1 ± 0.59) genes expression compared to untreated Panc-1 PBS (Fig. 7B, C, and D). C5EOSEW5050ESA treatment also resulted in the up-regulation of Notch target genes in Panc-1 PBS. However, C5EOSEW5050ESA significantly reduced the expression of HES-1 (3 ± 0.67), HEY-2 (1.5 ± 0.55), and Notch 1 (2 ± 0.57) genes in Panc-1 Gem compared to untreated Panc-1 Gem, denoting the ability of C5EOSEW5050ESA to hamper the Notch signalling pathway through the Notch 1 receptor that further inhibited the downstream Notch target genes including HES-1, HEY-2, and Notch-1 in Panc-1 Gem compared to the Panc-1 PBS (Fig. 7B, C and D).

### 3.9 C5EOSEW5050ESA induced cell death in gemcitabine-resistant cells

**Fig. 8:**
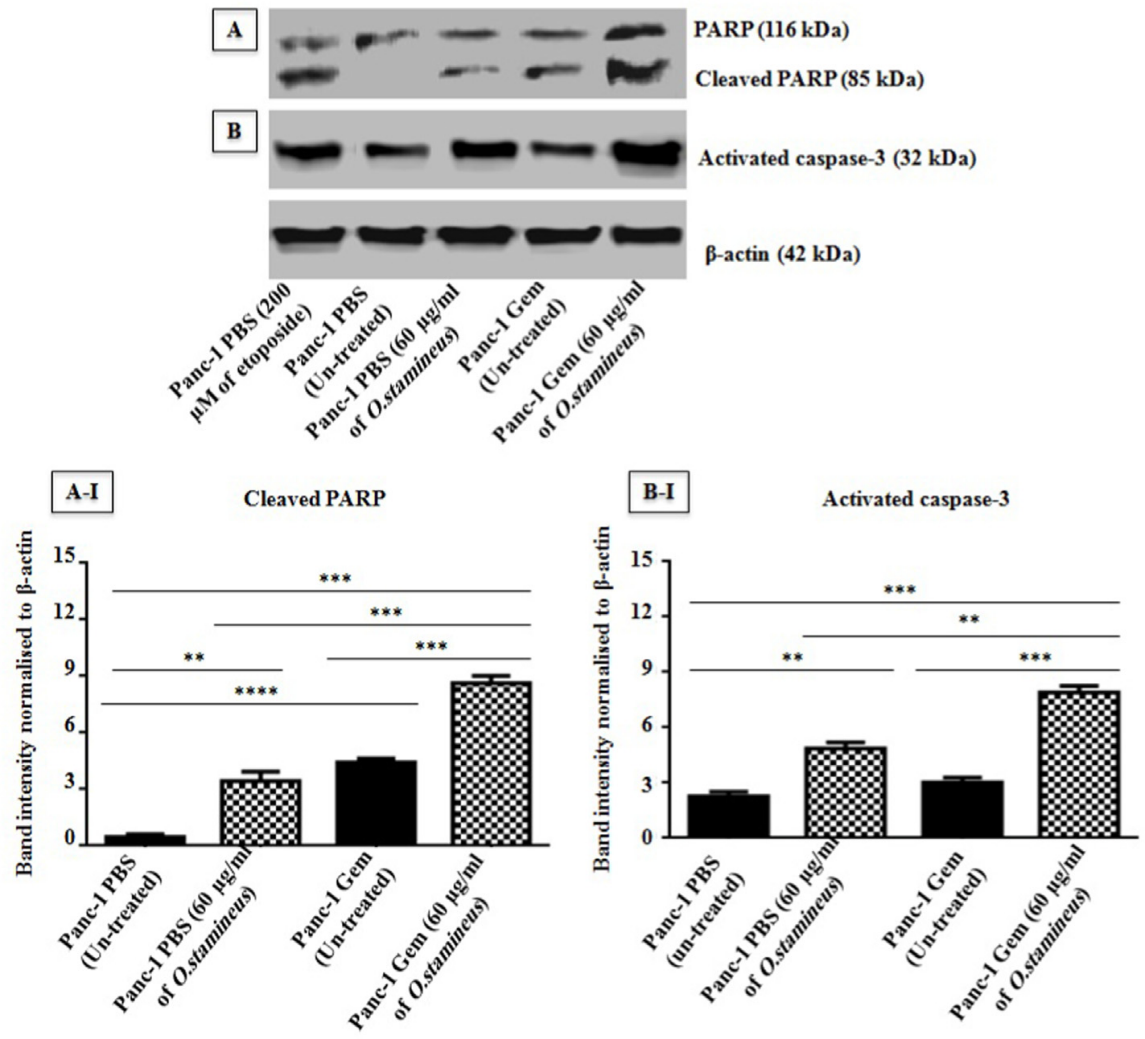
Protein expression profile of PARP and activated caspase-3 in resistant cells at 72 h post-treatment with *O. stamineus* (C5EOSEW5050ESA). Etoposide was used as a positive control. A) Cleaved PARP and B) activated caspase-3 proteins expression profiles were investigated through Western blot. Error bars represent SD. Statistics analysis (∗∗*P* < 0.01; ∗∗∗*P* < 0.001, One way ANOVA with Tukey's HSD post-hoc test, n = 3 independent experiments) using GraphPad Prism 6.0 software.

To examine the effect of C5EOSEW5050ESA on cell death, regulation of molecular markers was determined through Western blot, and Annexin V-PE and PI double staining were employed to detect the apoptotic and necrotic fractions in the resistant cells at 72 h post-treatment.Cleaved PARP and activated caspase-3 proteins were up-regulated in Panc-1 Gem compared to Panc-1 PBS cells by 1.1 ± 0.05 and 3 ± 0.03, respectively (Fig. 8). C5EOSEW5050ESA significantly up-regulated cleaved PARP and activated caspase-3 proteins by 3.4 ± 0.71 and 2.6 ± 0.44 respectively in Panc-1 PBS cells compared to untreated Panc-1 PBS cells. However, C5EOSEW5050ESA up-regulated cleaved PARP (8 ± 0.68) and activated caspase-3 (6 ± 0.71) proteins in Panc-1 Gem resistant cells compared tountreated Panc-1 Gem cells (Fig. 8).

**Fig. 9:**
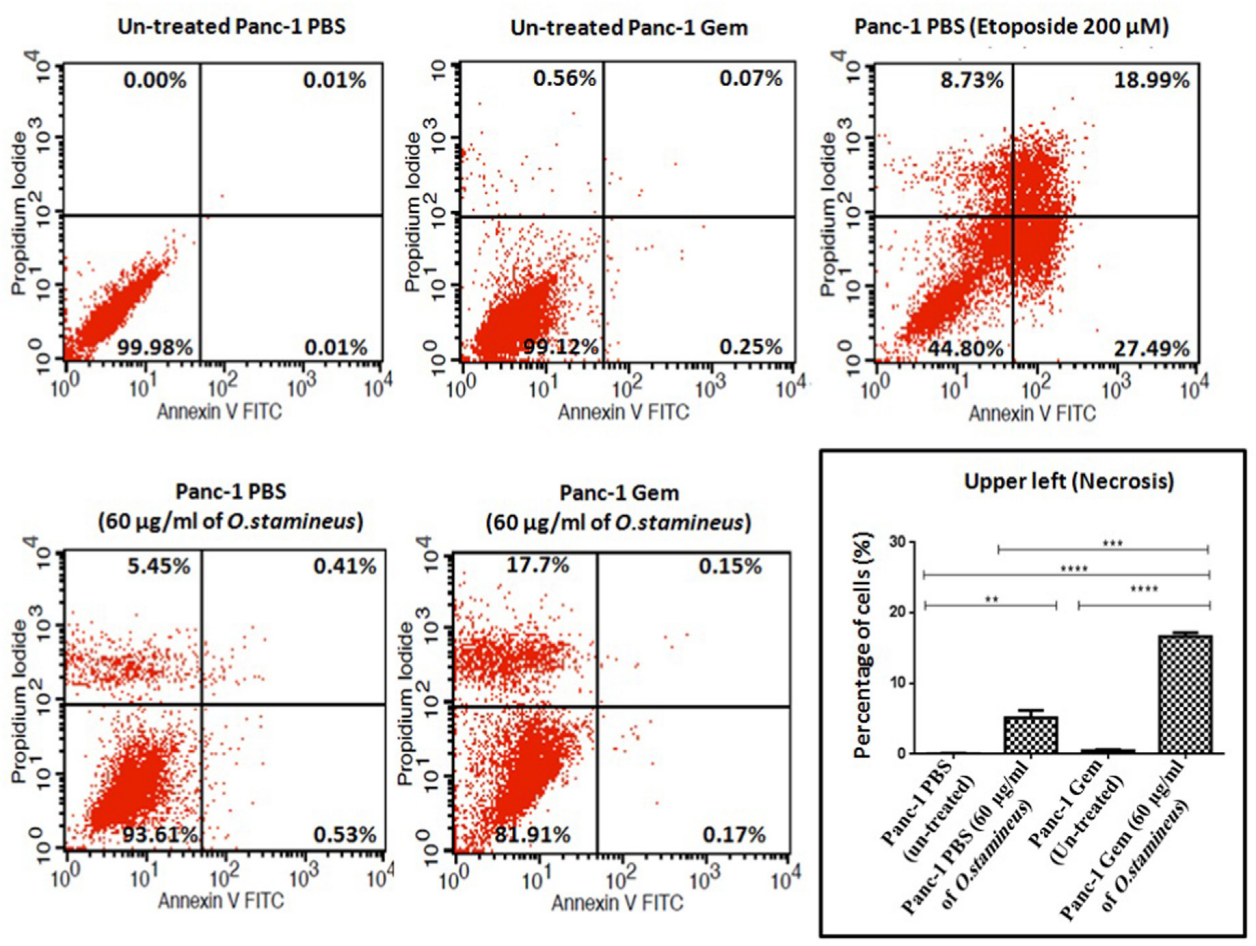
Flow cytometry analysis of cell death in gemcitabine-resistant Panc-1 cells induced by *O. stamineu* at 72 h post treatment. Results were expressed as the percentage of total cells. Cytogram showed lower left (live cells), upper left (necrotic cells), upper right (late apoptotic cells) and lower right (early apoptotic cells). Error bars represent SD. Statistics analysis (∗∗P < 0.01; ∗∗∗P < 0.001, ∗∗∗∗P < 0.0001 One way ANOVA with Tukey's HSD post-hoc test, n = 3 independent experiments) using GraphPad Prism 6.0 software.

Flow cytometry analysis revealed C5EOSEW5050ESA at the tested dose to significantly induce necrosis (17.7%) in Panc-1 Gem cells compared to treated Panc-1 PBS (5.45%) and untreated Panc-1 PBS (0.0%) and Panc-1 Gem (0.56%) cells (Fig. 9). The results revealed that C5EOSEW5050ESA significantly induced necrosis in Panc-1 Gem compared to untreated Panc-1 PBS and Panc-1 Gem. Interestingly, no apoptotic fractions were considerably detectedin C5EOSEW5050ESA-treated cells in both Panc-1 PBS and Panc-1 Gem groups, compared to their untreated groups. Etoposide induced necrosis and apoptosis in the cells.

## 4 Discussion

The current study showed CEOSEW5050ESA synergistically reduced cell viability of gemcitabine-resistant Panc-1 cells compared to untreated Panc-1 PBS and Panc-1 Gem cells (Fig. 1A). Although gemcitabine reduced the cell viability of Panc-1, Panc-1 PBS, and CCD841 cells (Fig. 1), the reduction was far less evident in CCD841 normal colon epithelial cells. Interestingly, C5EOSEW5050ESA exhibited no cytotoxic effect on CCD841 normal colon epithelial cells viability as the lethal dose was demonstrated to be a higher IC_50_ compared to resistant cells.This could be due to differences in doubling times between each cell line. The doubling time of Panc-1 and CCD841 cells has been reported to be 52 h and seven days, respectively.^16^^,^^17^ Eukaryotic cell division undergoes multiple convergent pathways to control genome duplicationonce during every cell division. When these pathways are devastated, random re-initiation of DNA replication occurs throughout the genome before mitosis, an event referred to as DNA re-replication. This gives rise to cells having greater than 4 N DNA (N designates the haploid DNA content of the genome) content that is susceptible to drugs that inactivate DNA damage response pathways.^18^ Although Panc-1 PBS cells were derived from Panc-1 cells, injected subcutaneously into mice, and treated with PBS over time, the IC_50_ values obtained (Fig. 1A) showed that Panc-1 PBS responded differently to treatments compared to Panc-1 cells, possibly due to changes affected by the tumour microenvironment after Panc-1 cells were injected subcutaneously into mice. Another study has corroborated this finding in which *O. stamineus* reduced the viability of prostate (PC3), breast (MDA-MB-231), and colon (HCT116) cancer cell lines compared to normal fibroblast cells.^19^

Eupatorin, sinesitin, and rosmarinic acid are the main component of C5EOSEW5050ESA. Eupatorin reduced the viability of Panc-1 Gem compared to rosmarinic acid and sinesitin (Fig. 1E, F, and G). Eupatorin has been reported to exhibit anti-proliferative activity on several cancer cell lines, including human gastric adenocarcinoma, human cervical adenocarcinoma, murine melanoma, murine colon carcinoma, and human breast cancer cell line.^20^

In addition, C5EOSEW5050ESA at 120 μg/ml shrank the spheroids compared to untreated Panc-1 PBS spheroids (Fig. 2). The decrease in spheroid size could be due to the induction of apoptosis, necrosis, and the reduction of cell viability of gemcitabine resistant cells. Flavonoids can induce necrosis and inhibit the invasion of chemoresistant cancer cells nuclear factor kappa B (NF-kB).^21^ Therefore, the reduction of spheroid size could be due to the flavonoid content of C5EOSEW5050ESA that induced cell death and reduced cell viability ofgemcitabine-resistant cells.

In cancer cells, senescence can be induced through DNA damage and overexpression of cell cycle inhibitor proteins. It has been demonstrated that gemcitabine could cause DNA damage in addition to inducing senescence in cancer cells.^22^ C5EOSEW5050ESA triggered cellular senescence in Panc-1 Gem compared to untreated Panc-1 PBS (Fig. 3), as also corroborated by Oon et al., 2015.^14^ This could be due to the high concentration of phenolic compounds in C5EOSEW5050ESA that triggered senescence in Panc-1 Gem, resulting in the reduction of cell viability (Fig. 3). Polyphenol compounds such as caffeic acid are one of the active compounds in the C5EOSEW5050ESA extract. It has been shown to suppress human lung, skin, bladder, breast, and prostate cancer cell growth and colony formation through the promotion of cellular senescence.^23^ However, no additional effect on cell cycle arrest was detected in Panc-1 Gem cells post C5EOSEW5050ESA treatment compared to Panc-1 PBS cells (Fig. 4). This could be due to cell cycle arrest at different phases of C5EOSEW5050ESA in gemcitabine resistant cells that cancelled out the effect of gemcitabine in Panc-1 Gem cells as demonstrated by our study in which gemcitabine arrested cells in the S phase. However, natural products mainly indicate cell cycle arrestin the G2/M checkpoint,^24^ as also depicted by C5EOSEW5050ESA-induced arrest in the G2 phase in Panc-1 PBS cells.

Panc-1 Gem cells portrayed an increase in MDR-1 and MRP-4 gene expression compared to Panc-1 PBS cells, indicating an established resistance in Panc-1 Gem cells (Fig. 5). No difference in MDR-1 gene expression was observed in untreated Panc-1 and Panc-1 PBS post C5EOSEW5050ESA treatments at 60 and 80 μg/ml (Fig. 5A). However, C5EOSEW5050ESA at 60 and 80 μg/ml significantly reduced the expression of MDR-1 and MRP-4 genes in Panc-1 Gem compared to untreated Panc-1 Gem, indicating a reversal of partial resistance by C5EOSEW5050ESA through down-regulation of multi-drug resistance genes that were induced by gemcitabine. Stilbene is a polyphenol compound that can mitigate P-glycoprotein activity in human adenocarcinoma cells.^25^ Stilbene is one of the bioactive compounds in C5EOSEW5050ESA extract that could work in synergy with other active compounds in C5EOSEW5050ESA and reduce drug resistance proteins in gemcitabine-resistant cells.^26^

Eupatorin at 25 and 50 μM further increased MDR-1 gene expression, indicating propagation of the P-glycoprotein resistance pump resulting in decreased intracellular accumulation of eupatorin (Fig. 5C). However, at both concentrations, eupatorin significantly reduced MRP-4 gene expression in Panc-1 Gem cells compared to untreated cells, indicating a partial reversal of resistance by eupatorin to gemcitabine (Fig. 5D). Previous studies have shown that cytochrome enzymes and transport-associated proteins especially MDR-1 play complementary parts in the induction of drug resistance in resistant cell lines.^27^ Eupatorin has demonstrated an ability to induce cytochrome P450 enzymes (CYPs) which are highly expressed in tumour cells.^28^ Hence, in the current study eupatorin could induce CYPs expression in gemcitabine-resistant cells that triggered MDR-1 gene expression in Panc-1 Gem cells, although this needs to be further investigated (Fig. 5C).

C5EOSEW5050ESA reduced the expression of Snail-1 and ZEB-1 genes in Panc-1 Gem compared to untreated and treated Panc-1 PBS cells (Fig. 6A and B). Besides, C5EOSEW5050ESA reduced vimentin protein expression compared to untreated and treated Panc-1 PBS cells and induced E-cadherin protein expression in Panc-1 Gem compared to untreated and treated Panc-1 PBS (Fig. 6C and D). Eupatorin induced EMT markers expression in gemcitabine-resistant cells (Fig. 6A and B). Our study showed that C5EOSEW5050ESA has a better effect than the bioactive compound, eupatorin. This could be due to polyphenolic compounds found in C5EOSEW5050ESA extract, which could enhance C5EOSEW5050ESA activity to reduce EMT markers in gemcitabine Panc-1 resistant cells. Many studies have shown that EMT is an essential regulator of ATP-binding cassette (ABC) transporter. A study by Tomono et al. showed that Snail-Induced EMT enhanced P-glycoprotein-mediated multi-drug resistance in lung adenocarcinoma cells.^29^

Notch 1 was previously reported to be elevated in pancreatic cancer and represents a potential target in pancreatic cancer.^30^ C5EOSEW5050ESA induced HEY-2 and Notch-1 genes in Panc-1 PBS compared to untreated (Fig. 7). In contrast, C5EOSEW5050ESA down-regulated the expression of Notch1 ICD protein and HEY-2, HES-1, and Notch-1 genes in Panc-1 Gem cells compared to untreated Panc-1 Gem cells, indicating the capability of C5EOSEW5050ESA to hamper the Notch signalling pathway through the Notch 1 receptor and Notch target genes. In pancreatic cancer, the Notch signalling pathway has been associated with increased survival and invasive properties of pancreatic cancer cells^31^ as such; C5EOSEW5050ESA could reduce Panc-1 Gem cell viability through inhibition of the Notch signalling pathway. MRK 003 is a γ-secretase inhibitor**,** demonstrated to down**-**regulate the Notch target genes**,** resulting in the induction of apoptosis and intra-tumoral necrosis and inhibition of tumo**u**r growth as well as increasing gemcitabine sensitivity in patient-derived pancreatic ductal carcinoma xenografts.^32^

Cell death plays an essential role in the treatment of cancer as it is a common target of many cancer treatment strategies. Necrosis, a form of non-apoptotic programmed cell death has recently been shown to play an important role in cancer chemotherapy, whereas the apoptosis pathways converge in the activation of caspase3, a downstream terminator caspase to induce cancer cell death.^33^Activated caspase-3 cleaves proteins, including poly (ADP-ribose) polymerase-1 (PARP-1), important in DNA repair to trigger apoptosis. Over-activation of PARP-1 may deplete nicotinamide adenine dinucleotide) (NAD^+^) and adenosine triphosphate (ATP), resulting in necrosis.^33^ The unexpected occurrence of necrosis over apoptosis despite caspase-3 activity incriminates a multifaceted mechanism controlling the verdict between both cell death pathways following C5EOSEW5050ESA treatment. Catalytically active Receptor Interacting Protein 1 (RIP1)-dependent programmed necrosis has previously beenreported to commit the cells to necrosis over apoptosis in the presence of caspase activity.^34^ Cleaved PARP and activated caspase-3 proteins were up-regulated in Panc-1 Gem compared to Panc-1 PBS cells (Fig. 8). C5EOSEW5050ESA up-regulated cleaved PARP and activated caspase-3 proteins in Panc-1 PBS cells compared to untreated Panc-1 PBS cells. However, C5EOSEW5050ESA induced cleaved PARP and activated caspase-3 proteins in Panc-1 Gem resistant cells compared to the untreated Panc-1 Gem cells. Remarkably, C5EOSEW5050ESA induced necrosis in Panc-1 Gem compared to untreated Panc-1 PBS and Panc-1 Gem cells (Fig. 9). C5EOSEW5050ESA was previously shown to induce necrosis in breast cancer xenograft model.^35^ In this study, C5EOSEW5050ESA triggered necrotic cell death in gemcitabine-resistant cells, potentially due to its cocktail of polyphenolic components that could also induce caspase-3 expression,^35^ highlighting the importance of dissecting the exact mechanism of action conferring to cell death induced by C5EOSEW5050ESA in gemcitabine-resistant cells in future studies.

Altogether, these imply that C5EOSEW5050ESA may confer gemcitabine-resistant pancreatic cancer cells to necrotic cell death through the blockade of the Notch signalling pathway by downregulating Notch1 ICD, HES-1, and HEY-2 to potentiate gemcitabine sensitivity in resistant pancreatic cancer cells.

## 5 Conclusions

This study demonstrates that the *O. stamineus*-derived extract C5EOSEW5050ESA present *in* Nuvastatic™ sensitised Panc-1 gemcitabine resistant cells to treatment by modulating the mechanisms and pathways that are involved in the chemo-sensitivity of gemcitabine resistance. The reduction of multi-drug resistance expression ingemcitabine-resistant cells is considered one of the major challenges. The impediment of the Notch signalling pathway through Notch 1 and its downstream components is postulated to be responsible for observed anti-cancer attributes of current chemo-herbal combinations. Moreover, eupatorin regulated some molecular players differently from C5EOSEW5050ESA, including MDR-1 and EMT. However, eupatorin failed to show any beneficial effects in gemcitabine-resistant cells, highlighting the potential advantages of botanical drugs in their polymolecular form instead of their constituents, in which the presence of other components may further enhance their efficacy. Therefore, it is concluded that C5EOSEW5050ESA can increase the effectiveness of chemotherapy treatment and improve clinical outcomes in patients with demonstrated gemcitabine resistance. These findings are valuable to support future clinical studies using Nuvastatic™ as an adjuvant treatment for pancreatic cancer patients receiving gemcitabine.

## Authors’ contributions

Ashwaq H.S. Yehya carried out the experiments and wrote the manuscript. Muhammad Asif conceived and analysed data. Amin M.S. Abdul Majid conceived and planned the experiments. Chern E. Oon designed, derived the model, supervised the project, proofread and provided critical feedback during manuscript writing and revision.

## Funding

This work was supported by the TWAS-USM fellowship and the National Key Economic Area (NKEA) Grant by the Ministry of Agriculture Malaysia (304/CIPPM/650736/k123).
